# Mr. Bush's Disavowal of All Connexion with the Cases Published by Mr. Yeatman in the Journal for July Last

**Published:** 1822-08

**Authors:** 


### Art. V.—Mr. Bush's *Disavowal of all Connexion with the Cases published by* Mr. Yeatman *in the Journal for July last.*

Last month, Mr. Yeatman favoured the world with "Observations on severely-contused Lacerated Wounds:" they are designed for the instruction of those who are not well-grounded in their profession! They are amusing, if not instructive. Lest they should appear to be given in illustration of the surgical practice of the "great medical authority" alluded to by him in the Journal for December,† I beg to disclaim all knowledge and connexion with his first and second cases; and to observe, all I had to do with his third, that of John Curtis, is given by me in the Journal for May.

*Frome; June 8th,* 1822.

